# Urinary matrix Gla protein is associated with mortality risk in Flemish population: A prospective study

**DOI:** 10.3389/fcvm.2022.894447

**Published:** 2022-07-22

**Authors:** Dongmei Wei, Jesus Melgarejo, Thomas Vanassche, Lucas Van Aelst, Stefan Janssens, Peter Verhamme, Zhen-Yu Zhang

**Affiliations:** ^1^Studies Coordinating Center, Research Unit Hypertension and Cardiovascular Epidemiology, KU Leuven Department of Cardiovascular Sciences, University of Leuven, Leuven, Belgium; ^2^Division of Cardiology, University Hospitals Leuven, Leuven, Belgium

**Keywords:** renal function, mortality, population science, biomarker, matrix Gla protein

## Abstract

**Background:**

Vascular calcification is strongly related to the risk of mortality and cardiovascular (CV) diseases. In vascular calcification, matrix Gla protein (MGP), a small vitamin K-dependent protein, is an important mineralization inhibitor. Recent studies showed that circulating MGP is associated with mortality risk. However, the longitudinal association between urinary excretion of MGP and all-cause mortality was not established.

**Materials and methods:**

Urinary MGP was measured in 776 randomly recruited Flemish population (mean age: 51.2 years; 50.9% women) at baseline (during 2005–2010) using capillary electrophoresis coupled with mass spectrometry. Plasma inactive MGP [desphospho-uncarboxylated MGP (dp-ucMGP)] levels were quantified in 646 individuals by ELISA kits. Mortality status was ascertained through the Belgian Population Registry until 2016. The longitudinal association with mortality was determined by the multivariate-adjusted Cox proportional hazards regression models. The multivariate linear regression models were used to identify determinants of urinary MGP level.

**Results:**

Over the 9.2 years, 47 (6.06%) participants died, including 15 CV deaths. For a doubling of urinary MGP, the hazard ratios (HRs) were 1.31 (95% CI: 1.01–1.69, *P* = 0.040) for all-cause mortality and 2.05 (95% CI: 1.11–3.79, *P* = 0.023) for CV mortality with adjustment for covariates, including estimated glomerular filtration rate and urine microalbumin. The addition of urinary MGP to the basic models improved the reclassification as suggested by the increased net reclassification improvement [64.01% (95% CI: 32.64–98.63)] and integrated discrimination improvement [2.33% (95% CI: 0.24–4.71)]. Circulating inactive MGP, total cholesterol, urine microalbumin, and smoking were significantly associated with urinary MGP levels (*P* ≤ 0.041), independent of sex and age.

**Conclusion:**

Elevated urinary MGP was associated with an increased risk of all-cause mortality and CV mortality and improved the risk reclassification for all-cause mortality. These findings suggested that urinary MGP might be useful in mortality risk assessment in the general population. However, these observations need to be replicated in larger studies with a longer follow-up time.

## Introduction

Vascular calcification that compromises arterial compliance and elastance and deteriorates cardiovascular hemodynamics is increasingly prevalent with advancing age, which is associated with adverse clinical impacts, such as congestive heart failure, aortic stenosis, and atherosclerotic plaque stability (1). Epidemiological studies have shown that vascular calcification is a strong risk factor for mortality and cardiovascular diseases (2–5). In vascular calcification, matrix Gla protein (MGP), a vitamin K-dependent protein, is a vital mineralization inhibitor that protects the arterial wall against calcium-binding and calcium crystallization (6, 7). Since the active status is a prerequisite for exerting the full inhibitory function of MGP, elevated circulating inactive MGP generally indicates progressive vascular calcification (8). Recent findings have repeatedly demonstrated that this circulating inactive MGP is associated with all-cause mortality, cardiovascular risk, regardless of the general population, type 2 diabetes, and vascular diseases (8–11).

The molecular weight of MGP is 11 kDa, whereas the glomerular filtration threshold is around 30–50 kDa, which enables MGP to freely filter through the glomerulus into urine (12). Urine is an easily accessible, stable, and non-invasive obtained biofluid that reserves abundant endogenous peptides and low-molecular-weight proteins derived from larger precursor proteins and protein degradation (13). Urinary proteins have been suggested to be indicative of diverse diseases, such as heart failure, coronary vasculopathy after heart transplantation, and chronic kidney disease (14–16). Considering the biological function of MGP and the merits of urinary biomarkers, it is clinically relevant to assess whether urinary MGP is associated with mortality. Determining this association not only promotes biomarker discovery for vascular calcification but also facilitates risk stratification and intervention for mortality. The interpretation of urinary MGP might be affected by multiple factors, thus it is also necessary to investigate the potential determinants, such as sex, age, renal function, and vitamin K status. The primary purpose of this study was to evaluate the association between urinary MGP and all-cause mortality in a general population with 9 years of follow-up. The secondary objective was to investigate the determinants of urinary MGP.

## Materials and methods

### Participants

This study was nested in a family-based study, the Flemish Study on Environment, Genes and Health Outcomes (FLEMENGHO), which enrolled individuals from 1985 until 2004 with an initial participation rate of 78% (17–19). The studies involving human participants were reviewed and approved by the University of Leuven Ethics Committee (17–19). The participants provided their written informed consent to participate in this study. Participants were repeatedly followed-up. From May 2005 until May 2010, 804 participants provided a 5 ml fresh middle-urine sample for urinary protein analysis. Of 799 participants with a valid measurement of urinary MGP, 23 participants were excluded because the interval between the biochemistry tests and urine sampling was over 1 month. Hence, this study included 776 participants, of whom 646 participants were measured with plasma inactive MGP in the meanwhile.

### Urinary matrix Gla protein (MGP) measurement

Urinary MGP was quantified by capillary electrophoresis coupled with mass spectrometry performed with a P/ACE MDQ CE (Beckman Colter, Fullerton, California, United States). coupled to a micro-TOF-MS (Bruker Daltonics, Bremen, Germany). MosaiquesVisu software was applied to process mass spectral data and generated peptides with molecular mass, migration time, and signal intensity that were calibrated using internal urinary standard peptides to assure the comparability between different datasets (20, 21). The peptides detected were assigned to the previously sequenced peptides from the Human Urinary Proteome Database by fragmenting peptides and matching the fragmentation spectra to the protein sequences from the databases such as the International Protein Index, the Reference Sequence Database at the National Center for Biotechnology Information (NCBI), and the UniProt Knowledgebase (22). Posttranslational modifications and specific mass spectra were considered when protein was annotated. Peptides from different samples were considered the same when the deviations of their molecular weight and the migration time were < 100 parts/million and < 1 min, respectively. Detailed information on sample preparation, proteome analysis, data processing, and sequencing of the peptides has been described in previous publications and the methods section of the supplements (23, 24).

### Biochemical measurements

Circulating inactive MGP [desphospho-uncarboxylated MGP (dp-ucMGP)], a surrogate for vitamin K status, was measured in citrated plasma samples by sandwich ELISA kits. This approach was developed by VitaK (Maastricht University, the Netherlands), which provided low variations of intra-assay (5.6%) and interassay (9.9%) with a minimum detection limit of 0.22 μg/l (25). The venous blood samples were obtained after at least 8 h of fasting. The measurement of lipid parameters and plasma glucose was performed with automated methods in certified laboratories. Serum creatinine was measured using the isotope-dilution mass spectrometry-traceable creatinine assay. The estimated glomerular filtration rate (eGFR) was calculated using the chronic kidney disease Epidemiology Collaboration creatinine equation (26). Participants collected a timed 24-h urine sample for the measurement of microalbumin.

### Clinical measurements

Hypertension was referred to as office systolic blood pressure ≥ 140 mm Hg or diastolic blood pressure ≥ 90 mm Hg or the prescription of antihypertensive drugs. Diabetes mellitus was defined as fasting blood glucose of ≥ 126 mg/dl or the use of antidiabetic drugs. History of smoking indicated inhaling tobacco daily in the past. Body mass index (BMI) was calculated by weight in kilograms divided by height in meters squared. Chronic kidney disease (CKD) was staged as 1 to 5 based on eGFR according to the guidelines from the National Kidney Foundation-Kidney Disease Outcomes Quality Initiative (27).

### Assessment of the outcomes

The vital status of all the participants was annually ascertained through the Belgian Population Registry in Brussels until 31 December 2016. In cases of death, the causes were indicated with the International Classification of Diseases (ICD) codes that were acquired from the Flemish Registry of Death Certificates. Physicians ascertained the diseases reported on the death certificates. Participants were censored when deaths occurred.

### Statistics

Data analysis was performed with SAS software, version 9.4 (SAS Institute, Cary, North Carolina, United States). Urinary MGP, plasma inactive MGP, and urine microalbumin were normalized by the transformation of the logarithm to base 2. Means and proportions were compared by the *t*-test, ANOVA, or Fisher’s exact test, as appropriate. Statistical significance was a two-sided *P*-value of 0.05.

In survival analysis, participants were grouped by one-third of urinary MGP. The crude cumulative incidence of all-cause mortality was estimated across the groups by the Kaplan–Meier method. The cumulative incidence curves were compared using the log-rank test. The associations of urinary MGP with all-cause mortality and cardiovascular mortality were assessed using the multivariable-adjusted Cox proportional hazards regression models. The proportional hazard assumption was examined by the Kolmogorov-type supremum test. According to previous publications and clinical relevance, potential confounders included age, sex, BMI, systolic blood pressure, diastolic blood pressure, smoking, diabetes mellitus, total cholesterol, low-density lipoprotein cholesterol, history of diabetes mellitus, history of cardiovascular diseases, eGFR, and urine microalbumin. The prognostic value for 8-year all-cause mortality added by urinary MGP was evaluated by the integrated discrimination improvement (IDI) and the net reclassification improvement (NRI) (28, 29). The IDI denoted the difference in discrimination slopes before and after adding the urinary MGP or dp-ucMGP into a reference model. The continuous NRI was calculated by 2 × (the percentage of cases with increased predicted probability – the percentage of non-cases with increased predicted probability) after adding urinary MGP. The 95% CIs and *P*-values for the NRI and the IDI were calculated by 500 times bootstrap methods.

When investigating the determinants of urinary MGP, the evaluated variables included sex, age, BMI, systolic blood pressure, diastolic blood pressure, smoking, diabetes mellitus, total cholesterol, low-density lipoprotein cholesterol, history of diabetes mellitus, history of cardiovascular diseases, eGFR, urine microalbumin, and circulating inactive MGP. Their associations with urinary MGP were primarily investigated with the univariate linear regression models. All these variables were added in the stepwise linear regression with backward selection and the variables that remained significant (*P* < 0.05) were considered determinants for urinary MGP. The collinearity of linear regression models was examined. The correlation coefficients and 95% CI were estimated by the linear regression model.

## Results

### Baseline characteristics

The age (SD) of 776 participants averaged 51.2 (15.7) years and 395 (50.9%) were women. The mean value for systolic/diastolic blood pressure was 129.6 (17.7)/79.7 (9.7) mm Hg, 26.5 (4.3) kg/m^2^ for body mass index, and 5.2 (1.0) mmol/l for total cholesterol. Of all the participants, 333 (42.9%) participants had hypertension, 34 (4.4%) participants had a history of diabetes mellitus, 62 (8.0%) participants experienced previous cardiovascular diseases, and 7 (0.9%) participants received treatment with warfarin. Of those with hypertension, 205 (61.6%) participants received antihypertensive treatment. The average eGFR was 85.1 (17.5) ml/min/1.73 m^2^ and 49 (6.3%) participants had an eGFR ≤ 60 ml/min/1.73 m^2^, including 48 at CKD stage 3 and 1 at CKD stage 4. In 776 participants, the median for urinary MGP level was 1,713 [interquartile range (IQR): 947–2,961] unit. Of 646 participants measured with plasma dp-ucMGP, the median of dp-ucMGP was 0.42 (IQR: 0.28–0.58) nmol/l. Table 1 shows the baseline characteristics of participants by one-third of the distribution of urinary MGP. The characteristics of participants across the tertile of plasma dp-ucMGP are given in Supplementary Table 1.

**TABLE 1:** Participant characteristics and classified according to one-third of urinary matrix Gla protein (MGP).

Characteristics	Low (*n* = 258)	Medium (*n* = 259)	High (*n* = 259)	*P* for trend
Urinary MGP (Log_2_), unit	<10.21	10.21–11.27	>11.27	
Number with characteristic (%)				
Female	116 (45.0)	130 (50.2)	149 (57.5)	0.004
Current Smoking	61 (23.6)	47 (18.2)	43 (16.6)	0.043
Current alcohol intake	187 (72.5)	183 (70.7)	167 (64.5)	0.049
Diabetes mellitus	13 (5.0)	9 (3.5)	12 (4.6)	0.82
History of CVD	8 (3.1)	18 (7.0)	36 (13.9)	<0.0001
Hypertension	80 (31.0)	109 (42.1)	144 (55.6)	<0.0001
Treatment of hypertension	51 (19.8)	62 (23.9)	92 (35.5)	0.0001
Statins	32 (12.4)	27 (10.4)	42 (16.2)	0.20
Warfarin	0 (0)	0 (0)	7 (2.7)	0.001
Mean (± SD) or median (IQR)				
Age, years	44.7 ± 16.5	51.2 ± 14.0	57.5 ± 13.8	<0.0001
Body mass index, kg/m^2^	25.4 ± 4.2	26.9 ± 4.4	27.1 ± 4.3	<0.0001
Waist-to-hip ratio	0.85 ± 0.09	0.88 ± 0.08	0.89 ± 0.08	<0.0001
Systolic blood pressure, mmHg	124.3 ± 15.1	129.7 ± 17.3	134.8 ± 19.0	<0.0001
Diastolic blood pressure, mmHg	77.7 ± 10.3	80.8 ± 9.2	80.7 ± 9.2	0.0002
Serum total cholesterol, mmol/L	4.99 ± 0.99	5.28 ± 0.88	5.46 ± 0.99	<0.0001
HDL-cholesterol, mmol/L	1.42 ± 0.37	1.40 ± 0.33	1.45 ± 0.36	0.19
LDL-cholesterol, mmol/L	2.98 ± 0.86	3.26 ± 0.77	3.37 ± 0.87	<0.0001
Blood glucose, mmol/L	4.91 ± 1.01	4.95 ± 0.74	4.95 ± 0.57	0.015
Serum creatinine, mg/dL	0.91 ± 0.15	0.93 ± 0.17	0.94 ± 0.21	0.39
eGFR, ml/min/1.73m^2^	91.4 ± 17.8	84.4 ± 16.2	79.5 ± 16.5	<0.0001
Urine albumin, mg/L	5.10 (3.50, 7.40)	5.50 (4.10, 7.30)	6.00 (4.60, 8.50)	<0.0001
Plasma dp-ucMGP, nmol/L	0.32 (0.22, 0.45)	0.43 (0.30–0.56)	0.53 (0.36, 0.69)	<0.0001

Current smoking refers to inhaling tobacco daily; Diabetes mellitus was use of antidiabetic drugs, fasting blood glucose of ≥ 126 mg/dL; Hypertension was an office blood pressure of ≥ 140 mmHg systolic or ≥ 90 mmHg diastolic, or use of antihypertensive drugs; Body mass index was calculated by weight in kilograms divided by height in meters squared; Glomerular filtration rate was estimated using the chronic kidney disease epidemiology collaboration creatinine equation.

CVD, cardiovascular diseases; eGFR, estimated glomerular filtration rate; HDL, high-density lipoprotein; IQR, interquartile range; LDL, high–density lipoprotein; MGP, matrix Gla protein; SD, standard deviation.

### Associations of mortality risk with urinary matrix Gla protein

**FIGURE 1:**
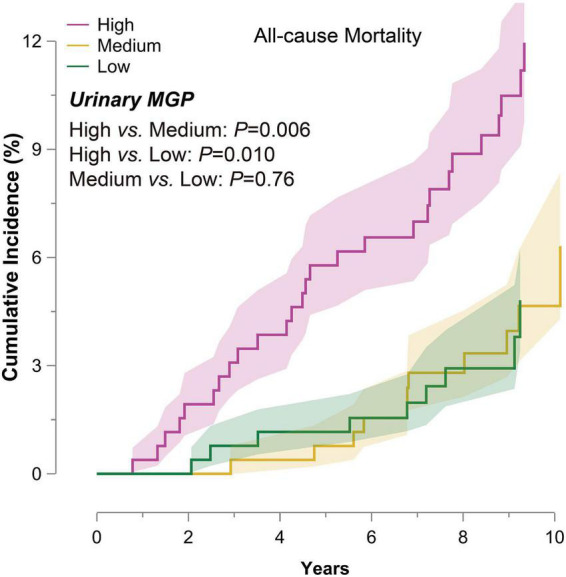
Cumulative incidence of all-cause mortality in 776 participants by one-third of urinary matrix Gla protein (MGP). The participants were divided into the three groups by one-third of urinary MGP. The colored bands represent SE. *P*-values express the significance of the log-rank test for the difference across one-third of urinary MGP. Abbreviation: MGP, matrix Gla protein; SE, standard error.

The median time of follow-up was 9.2 (5th–95th percentile, 5.8–10.7) years. Of 776 participants, there were 47 deaths (6.06%), including 15 cardiovascular deaths. As shown in Figure 1, the cumulative incidence of all-cause mortality at the top third of urinary MGP was significantly higher than those at the bottom third of urinary MGP [unadjusted HR = 2.90 (95% CI: 1.25–5.08), *P* = 0.006].

With the adjustment of age, sex, body mass index, systolic blood pressure, diastolic blood pressure, smoking, total cholesterol, low-density lipoprotein cholesterol, history of diabetes mellitus, and history of cardiovascular diseases, the risk of all-cause mortality was 1.31 (95% CI: 1.02–1.69, *P* = 0.006) for a doubling of urinary MGP (Table 2). A doubling of urinary MGP was still associated with a 31% increased risk of all-cause mortality [HR: 1.31 (95% CI: 1.01-1.69), P = 0.040] after further adjustment for eGFR and urine microalbumin. Similarly, higher urinary MGP was significantly associated with an increased risk of cardiovascular mortality. In the fully adjusted model, the risk of cardiovascular mortality was 2.05 (95% CI: 1.11–3.79, *P* = 0.023) for a doubling of urinary MGP. All the models met the proportional hazard assumption.

**TABLE 2:** Association of urinary matrix Gla protein (MGP) with all-cause mortality and cardiovascular mortality.

Outcomes	Events	Urinary MGP		
		HR	95% CI	*P*
All-cause mortality	47/776			
Crude		1.63	1.27–2.08	0.0001
Adjusted model 1		1.31	1.02–1.69	0.034
Adjusted model 2		1.31	1.01–1.69	0.040
Cardiovascular mortality	15/776			
Crude		2.38	1.51–3.77	0.0002
Adjusted model 1		2.04	1.14–3.65	0.017
Adjusted model 2		2.05	1.11–3.79	0.023

Adjusted model 1: adjusted for age, sex, body mass index, systolic blood pressure, diastolic blood pressure, smoking, total cholesterol, low-density lipoprotein cholesterol, history of diabetes mellitus, and history of cardiovascular diseases.

Adjusted model 2: adjusted for variables included by model 1 plus estimated glomerular filtration rate and urine microalbumin.

CI, confidence interval; HR, hazard ration; MGP, matrix Gla protein.

### Urinary matrix Gla protein (MGP) and all-cause mortality risk discrimination and reclassification

The utilization of urinary MGP in all-cause mortality risk reclassification was assessed by the NRI and the IDI (Table 3). The basic model comprised of common risk factors, including age, sex, BMI, systolic blood pressure, diastolic blood pressure, smoking, diabetes mellitus, total cholesterol, low-density lipoprotein cholesterol, history of diabetes mellitus, history of cardiovascular diseases, eGFR, and urine microalbumin. The categorical free NRI was 64.01% (95% CI: 32.64–98.63, *P* < 0.0001) overall, 37.88% (95% CI: 8.31–70.37, *P* = 0.018) in cases, and 26.14% (95% CI: 19.10–33.04, *P* < 0.0001) in controls. The IDI was 2.33% (95% CI: 0.24-4.71, *P* = 0.018).

**TABLE 3:** Improvements in risk discrimination and reclassification for all-cause mortality upon addition of urinary matrix Gla protein (MGP).

Index	Improvements		
	Estimate,%	95% CI	*P*
Net reclassification improvement	64.01	32.64–98.63	<0.0001
Net reclassification improvement _*event*_	37.88	8.31–70.37	0.018
Net reclassification improvement _*non–event*_	26.14	19.10–33.04	<0.0001
Integrated discrimination improvement	2.33	0.24–4.71	0.048

The basic models included age, sex, body mass index, systolic blood pressure, diastolic blood pressure, smoking, diabetes mellitus, total cholesterol, low-density lipoprotein cholesterol, history of diabetes mellitus, history of cardiovascular diseases, estimated glomerular filtration rate, and urine microalbumin. CI, confidence interval.

### Determinants of urinary matrix Gla protein (MGP)

In the unadjusted linear regression analysis, urinary MGP was significantly associated with all the variables given in Table 4, except for diabetes. eGFR was inversely associated with urinary MGP [coefficient: –0.022 (95% CI: –0.027 to –0.017)], whereas urine microalbumin was positively associated with urinary MGP [coefficient: 0.236 (95% CI: 0.149–0.324)]. However, the association of urinary MGP with eGFR became non-significant [coefficient: –0.004 (95% CI: –0.011 to 0.004)] when considering other variables, but the association with urine microalbumin remained. Similarly, CKD (≤ 60 ml/min/1.73 m^2^) was also not significantly associated with urinary MGP levels [coefficient: 0.020 (95% CI: –0.391 to 0.431)]. Another determinant of urinary MGP was circulating inactive MGP [coefficient: 0.149 (95% CI: 0.062–0.237)], regardless of other variables. In the final multivariate-adjusted linear regression model, urinary MGP was also significantly higher among women compared with men [coefficient: 0.370 (95% CI: 0.180–0.559)] (Table 4), while smokers had lower urinary MGP than non-smokers [coefficient: –0.255 (95% CI: –0.501 to –0.010)]. In addition, urinary MGP was significantly increased with age [coefficient: 0.012 (95% CI: 0.005–0.019)] and total cholesterol [coefficient: 0.372 (95% CI: 0.247–0.497)], as shown in Table 4. In addition, urinary MGP was significantly associated with urine creatinine [coefficient: –0.049 (95% CI: –0.069 to –0.029)] in the univariate analysis, but the association lost significance after adjusting for sex and age [coefficient: –0.008 (95% CI: –0.033 to 0.017), *P* = 0.53].

**TABLE 4:** Determinants of urinary matrix Gla protein (MGP).

Variables	Unadjusted model*		Adjusted model*		Final model	
	Coefficient	95% CI	Coefficient	95% CI	Coefficient	95% CI
Sex: Female	0.292†	0.104, 0.481	0.385^†^	0.185, 0.586	0.370^†^	0.180, 0.559
Smoking	–0.345^†^	–0.583, –0.107	–0.233	–0.479, 0.014	–0.255^†^	–0.501, –0.010
Diabetes	–0.123	–0.586, 0.339	–0.466	–0.945, 0.014		
History of CVD	0.764*	0.419, 1.109	0.312	–0.068, 0.692		
Age	0.027*	0.021, 0.032	0.007	–0.003, 0.017	0.012^†^	0.005, 0.019
BMI	0.049*	0.028, 0.071	0.016	–0.007, 0.040		
SBP	0.017*	0.012, 0.023	0.003	–0.005, 0.010		
DBP	0.015*	0.005, 0.025	0.001	–0.011, 0.013		
Total cholesterol	0.251*	0.155, 0.347	0.228	–0.032, 0.488	0.190^†^	0.087, 0.293
LDL cholesterol	0.261*	0.151, 0.371	–0.066*	–0.360, 0.228		
Inactive MGP	0.555*	0.439, 0.671	0.338	0.208, 0.467	0.372*	0.247, 0.497
eGFR	–0.022*	–0.027, –0.017	–0.004	–0.011, 0.004		
Urine microalbumin	0.236*	0.149, 0.324	0.146†	0.057, 0.235	0.149^†^	0.062, 0.237

*P < 0.0001; P < 0.001; ^†^P < 0.05.

Adjusted model indicated the multivariable linear regression model including all the listed variables.

Final model included the variables that remained significant in the stepwise linear regression model with the backward selection.

BMI, body mass index; CVD, cardiovascular diseases; CI, confidence interval; DBP, diastolic blood pressure; eGFR, estimated glomerular filtration rate; HDL, high-density lipoprotein; LDL, high–density lipoprotein; MGP, matrix Gla protein; SBP, systolic blood pressure.

## Discussion

Our main finding was that urinary MGP was associated with the increased risk of all-cause mortality and cardiovascular mortality, independent of renal function, urine microalbumin, and other clinical risk factors. Specifically, urinary MGP further improved the risk reclassification for all-cause mortality on the basis of known risk factors, which was supported by the increased NRI and IDI. The longitudinally results demonstrated that urinary MGP may be used as a novel marker for the risk stratification of all-cause mortality. The level of urinary MGP was suggested to be associated with sex, age, circulating inactive MGP, urine microalbumin, smoking, and total cholesterol, but not with eGFR.

The prospective association of urinary MGP and all-cause mortality is consistent with previous evidence on elevated MGP in circulation and mortality (8, 10, 11, 30–32). In a longitudinal study that included 2,318 Flemish population with 14.1 years of follow-up, a doubling of dp-ucMGP at baseline is associated with 15% increased risk of mortality (8). Similarly, another study on 799 patients experiencing coronary events or ischemic stroke showed that patients in the highest quartile of dp-ucMGP had a 1.89-fold risk of all-cause mortality and 1.88-fold cardiovascular mortality in a period of 5.6 years of follow-up (10). High dp-ucMGP level was significantly associated with short-term mortality in patients with severe calcific valvular aortic stenosis (11). Likewise, this association between circulating inactive MGP and mortality has been observed in patients with CKD or end-stage renal disease (ESRD) where vascular calcification is generally pronounced (30–32). Besides, dp-ucMGP has been suggested to be positively associated with established cardiovascular risk factors, such as systolic blood pressure, obesity, and pulse wave velocity (33). Indeed, the precursors of MGP experience two sequential posttranslational modifications, namely, γ-carboxylation and phosphorylation, before being fully functional, thus MGP exists in several species due to different phosphorylation and carboxylation status (25). In this study, the detected urinary MGP did not exclusively originate from dp-ucMGP, but from all the formations of MGP. Limited studies reported the association of total uncarboxylated MGP with adverse outcomes, though the results were inconclusive (9, 10, 34). Along with this epidemiological evidence, experimental data also suggested that MGP is necessary for maintaining normal vascular function. The MGP knockout mice (MGP -/-) died from excessive arterial calcification after 6–8 weeks after birth (35). The polymorphisms of the *MGP* gene were suggested to attribute to an increased risk of plaque calcification and myocardial infarction (36). To the best of our knowledge, this is the first study to investigate the association of urinary MGP with mortality in a general population. This informative MGP well exemplified that the property of small molecular weight allows circulating MGP to excrete through the kidney into urine and urinary MGP could be a useful marker.

The intuitive improvements in risk reclassification for all-cause mortality suggested that urinary MGP may facilitate the development of a novel framework for risk stratification and intervention. Particularly, urinary MGP seemed to capture the risk that was not covered by the common risk factors, such as age, cholesterol, and urine microalbumin. Several merits make urinary MGP a favorable choice for the prediction of mortality, including obtaining an abundant quantity of urine samples with a non-invasive approach, relatively stable proteins, and a detective method with high reproductivity.

Urinary MGP might be related to vitamin K status, as urinary MGP level was associated with circulating dp-ucMGP. As the γ-carboxylation of MGP is a vitamin K-dependent process, circulating dp-ucMGP is considered a surrogate for functional vitamin K status in clinical research (37). Due to suppressing MGP activation, poor vitamin K status is associated with deteriorated vascular calcification. Warfarin, for instance, is a vitamin K antagonist that is used as an oral anticoagulant in clinical practice. In patients receiving warfarin, the prevalence of arterial calcification on X-ray was 44% than in those patients without warfarin (38). More important, vitamin K supplementation seems to promote the activation of MGP, reduce dp-ucMGP level, and slow the progression of vascular calcification (39). Therefore, the appropriate assessment of vitamin K status could pave the way for the use of vitamin K supplements to improve the adverse outcomes. Given the association between urinary MGP and circulating dp-ucMGP, urinary MGP might be correlated with active vitamin K status, although future studies are required to investigate this association.

The finding of this study that eGFR was not associated with urinary MGP levels is consistent with a prior study on the renal excretion of MGP. In 90 participants with moderate-to-severe hypertension who underwent renal artery angiography, the renal fractional extraction of MGP was 12.8% and independent of renal function by comparing the arterial and venous MGP concentrations (40). Of note, even though creatinine clearance was in the wide range of 26–154 ml/min, the extraction fraction was neither correlated with serum creatinine nor endogenous creatinine clearance (40). Our study also showed that CKD status (≤ 60 ml/min/1.73 m^2^) appeared to not associate with urinary MGP as well. On the contrary, accumulating evidence has demonstrated that high dp-ucMGP is associated with renal dysfunction in multiple populations (41–44). For instance, Wei et al. confirmed the longitudinal association of dp-ucMGP with the risk of progression to CKD (eGFR < 60 ml/min/1.73 m^2^) and microalbuminuria after adjusting for baseline eGFR and other clinical variables in 1,009 Flemish population in a follow-up of 8.9 years (42). However, another study showed that the association of dp-ucMGP with the incidence of CKD failed to replicate in a prospective study of 3,639 healthy adults after adjustment for baseline eGFR (45). Considering the high prevalence of vitamin K deficiency among patients with CKD, the association between dp-ucMGP and renal function is likely caused by poor vitamin K status (46). Interestingly, urine microalbumin was proportionally increased with urinary MGP, reflecting the ability of a small protein to filter through the glomerulus, even with normal eGFR. Nonetheless, the association of urinary MGP with mortality was independent of urine microalbumin.

The finding in the present study that urinary MGP levels were increased with advancing age is in the same line of previous studies on dp-ucMGP and aging (8, 10, 45). This might be related to the degenerative vascular calcification in advancing age. Higher urinary MGP in women is also supported by the observation of elevated dp-ucMGP (8, 10). Smoking had an inverse association with urinary MGP, in consistent with less prevalent smoking in participants with high dp-ucMGP, although no published evidence on the mechanism of smoking and MGP expression (8, 10). The association of urinary MGP with cholesterol might involve the cholesterol deposition in atherosclerotic calcification (7).

The noteworthy strengths of this study included the well-characterized population to minimize the potential confounding, the prospective study design and long-term follow-up, and the assessment of the improvement in the discrimination and reclassification for all-cause mortality. Furthermore, the available data on inactive MGP, urine albumin, and eGFR helped to address the potential impact of renal function and vitamin K status on urinary MGP.

### Limitations

However, our findings must be interpreted within the context. It is noteworthy that although the association between urinary MGP and mortality risk was observed in our population, it needs to be further validated before generalizing to other large cohorts with longer exposure. As an observational study, there might be potential residual confounding factors, although various clinical risk factors were included to eliminate the potential confounding effects. Hence, causality cannot be confirmed by this observation. Besides, plasma vitamin K levels and food frequency questionnaires were not available to directly evaluate vitamin K status. However, the food frequency questionnaires that measure dietary intake and food compositions are prone to be affected by diverse lifestyles and cultures (37). Plasma vitamin K reflects the intake of vitamin K, rather than functional vitamin K, and it was not conventional in clinical practice. Alternatively, dp-ucMGP is considered a reliable marker for functional vitamin K status. Future studies are warranted to validate the association of urinary MGP with vitamin K status determined by plasma vitamin K levels and food frequency questionnaires. Furthermore, we did not measure the total MGP because circulating MGP has several species and no mature commercial kits for measurement of total MGP were developed. This present study only measured one isoform of MGP, dp-ucMGP, in plasma. Moreover, this study used the overall urinary MGP since the untargeted proteomics approach did not distinguish the different isoforms of MGP in urine. This might hinder the investigation of the constitution of urinary MGP and its association with plasma MGP species and adverse outcomes. Last, the majority of the studied population were Caucasian, thus it should be cautiously generalized to other ethnicities.

## Conclusion

This study underlined that urinary MGP was significantly associated with all-cause mortality and cardiovascular mortality. The inclusion of urinary MGP could improve the risk reclassification for all-cause mortality. Urinary MGP levels were associated with circulating inactive MGP, sex, age, and urine microalbumin but were not influenced by eGFR.

## Data availability statement

The original contributions presented in this study are included in the article/Supplementary Material, the datasets analyzed are not publicly available due to participants’ privacy protection but are available from the corresponding author upon reasonable request.

## Ethics statement

The studies involving human participants were reviewed and approved by the University of Leuven Ethics Committee. Written informed consent to participate in this study was provided by the participants’ legal guardian/next of kin.

## Author contributions

DW and Z-YZ were responsible for conceptualization. Z-YZ was responsible for funding acquisition and resources. Z-YZ, TV, LVA, SJ, and PV were responsible for supervision. DW and Z-YZ were responsible for analysis, data curation, and validation, writing – original draft. All authors revised the manuscript and approved the final version of the manuscript.

## Conflict of interest

The authors declare that the research was conducted in the absence of any commercial or financial relationships that could be construed as a potential conflict of interest.

## Publisher’s note

All claims expressed in this article are solely those of the authors and do not necessarily represent those of their affiliated organizations, or those of the publisher, the editors and the reviewers. Any product that may be evaluated in this article, or claim that may be made by its manufacturer, is not guaranteed or endorsed by the publisher.

## References

[1] Demer LL Tintut Y. Vascular calcification: pathobiology of a multifaceted disease. *Circulation.* (2008) 117:2938–48. 10.1161/CIRCULATIONAHA.107.743161 18519861 PMC4431628

[2] Chen J Budoff MJ Reilly MP Yang W Rosas SE Rahman M Coronary artery calcification and risk of cardiovascular disease and death among patients with chronic kidney disease. *JAMA Cardiol.* (2017) 2:635–43. 10.1001/jamacardio.2017.0363 28329057 PMC5798875

[3] Adragao T Pires A Lucas C Birne R Magalhaes L Goncalves M A simple vascular calcification score predicts cardiovascular risk in haemodialysis patients. *Nephrol Dial Transplant.* (2004) 19:1480–8. 10.1093/ndt/gfh217 15034154

[4] Malik S Zhao Y Budoff M Nasir K Blumenthal RS Bertoni AG Coronary artery calcium score for long-term risk classification in individuals with type 2 diabetes and metabolic syndrome from the multi-ethnic study of atherosclerosis. *JAMA Cardiol.* (2017) 2:1332–40. 10.1001/jamacardio.2017.4191 29117273 PMC5814996

[5] Bos D Leening MJ Kavousi M Hofman A Franco OH van der Lugt A Comparison of atherosclerotic calcification in major vessel beds on the risk of all-cause and cause-specific mortality: the Rotterdam study. *Circ Cardiovasc Imaging.* (2015) 8:e003843. 10.1161/CIRCIMAGING.115.003843 26659376

[6] Proudfoot D Shanahan CM. Molecular mechanisms mediating vascular calcification: role of matrix Gla protein. *Nephrology.* (2006) 11:455–61. 10.1111/j.1440-1797.2006.00660.x 17014561

[7] Johnson RC Leopold JA Loscalzo J. Vascular calcification: pathobiological mechanisms and clinical implications. *Circ Res.* (2006) 99:1044–59. 10.1161/01.RES.0000249379.55535.21 17095733

[8] Liu YP Gu YM Thijs L Knapen MH Salvi E Citterio L Inactive matrix Gla protein is causally related to adverse health outcomes: a mendelian randomization study in a Flemish population. *Hypertension.* (2015) 65:463–70. 10.1161/hypertensionaha.114.04494 25421980

[9] Dalmeijer GW van der Schouw YT Magdeleyns EJ Vermeer C Verschuren WM Boer JM Matrix gla protein species and risk of cardiovascular events in type 2 diabetic patients. *Diabetes Care.* (2013) 36:3766–71. 10.2337/dc13-0065 23877986 PMC3816877

[10] Mayer O Jr. Seidlerova J Bruthans J Filipovsky J Timoracka K Vanek J Desphospho-uncarboxylated matrix gla-protein is associated with mortality risk in patients with chronic stable vascular disease. *Atherosclerosis.* (2014) 235:162–8. 10.1016/j.atherosclerosis.2014.04.027 24835435

[11] Ueland T Gullestad L Dahl CP Aukrust P Aakhus S Solberg OG Undercarboxylated matrix gla protein is associated with indices of heart failure and mortality in symptomatic aortic stenosis. *J Intern Med.* (2010) 268:483–92. 10.1111/j.1365-2796.2010.02264.x 20804515

[12] Ruggiero A Villa CH Bander E Rey DA Bergkvist M Batt CA Paradoxical glomerular filtration of carbon nanotubes. *Proc Natl Acad Sci USA.* (2010) 107:12369–74. 10.1073/pnas.0913667107 20566862 PMC2901461

[13] Mischak H Julian BA Novak J. High-resolution proteome/peptidome analysis of peptides and low-molecular-weight proteins in urine. *Proteomics Clin Appl.* (2007) 1:792. 10.1002/prca.200700043 20107618 PMC2811330

[14] He T Mischak M Clark AL Campbell RT Delles C Diez J Urinary peptides in heart failure: a link to molecular pathophysiology. *Eur J Heart Fail.* (2021) 23:1875–87. 10.1002/ejhf.2195 33881206 PMC9291452

[15] Wei D Trenson S Van Keer JM Melgarejo J Cutsforth E Thijs L The novel proteomic signature for cardiac allograft vasculopathy. *ESC Heart Fail.* (2022) 9:1216–27. 10.1002/ehf2.13796 35005846 PMC8934921

[16] Schanstra JP Zurbig P Alkhalaf A Argiles A Bakker SJ Beige J Diagnosis and prediction of CKD progression by assessment of urinary peptides. *J Am Soc Nephrol.* (2015) 26:1999–2010. 10.1681/ASN.2014050423 25589610 PMC4520165

[17] Zhang ZY Thijs L Petit T Gu YM Jacobs L Yang WY Urinary proteome and systolic blood pressure as predictors of 5-year cardiovascular and cardiac outcomes in a general population. *Hypertension.* (2015) 66:52–60. 10.1161/HYPERTENSIONAHA.115.05296 26063667

[18] Wei FF Thijs L Yu CG Melgarejo JD Zhang ZY Maestre GE Retinal microvasculature in relation to central hemodynamics in a flemish population. *Hypertension.* (2019) 74:606–13. 10.1161/HYPERTENSIONAHA.119.13255 31280648 PMC6687036

[19] Wei FF Zhang ZY Thijs L Yang WY Jacobs L Cauwenberghs N Conventional and ambulatory blood pressure as predictors of retinal arteriolar narrowing. *Hypertension.* (2016) 68:511–20. 10.1161/HYPERTENSIONAHA.116.07523 27324224 PMC4956676

[20] Haubitz M Good DM Woywodt A Haller H Rupprecht H Theodorescu D Identification and validation of urinary biomarkers for differential diagnosis and evaluation of therapeutic intervention in anti-neutrophil cytoplasmic antibody-associated vasculitis. *Mol Cell Proteomics.* (2009) 8:2296–307. 10.1074/mcp.M800529-MCP200 19564150 PMC2758757

[21] Jantos-Siwy J Schiffer E Brand K Schumann G Rossing K Delles C Quantitative urinary proteome analysis for biomarker evaluation in chronic kidney disease. *J Proteome Res.* (2009) 8:268–81. 10.1021/pr800401m 19012428

[22] Stalmach A Albalat A Mullen W Mischak H. Recent advances in capillary electrophoresis coupled to mass spectrometry for clinical proteomic applications. *Electrophoresis.* (2013) 34:1452–64. 10.1002/elps.201200708 23512263

[23] Mischak H Kolch W Aivaliotis M Bouyssie D Court M Dihazi H Comprehensive human urine standards for comparability and standardization in clinical proteome analysis. *Proteomics Clin Appl.* (2010) 4:464–78. 10.1002/prca.200900189 21137064 PMC3064949

[24] Mischak H Vlahou A Ioannidis JP. Technical aspects and inter-laboratory variability in native peptide profiling: the CE-MS experience. *Clin Biochem.* (2013) 46:432–43. 10.1016/j.clinbiochem.2012.09.025 23041249

[25] Cranenburg EC Koos R Schurgers LJ Magdeleyns EJ Schoonbrood TH Landewe RB Characterisation and potential diagnostic value of circulating matrix gla protein (MGP) species. *Thromb Haemost.* (2010) 104:811–22. 10.1160/TH09-11-0786 20694284

[26] Stevens LA Claybon MA Schmid CH Chen J Horio M Imai E Evaluation of the chronic kidney disease epidemiology collaboration equation for estimating the glomerular filtration rate in multiple ethnicities. *Kidney Int.* (2011) 79:555–62. 10.1038/ki.2010.462 21107446 PMC4220293

[27] Improving Global Outcomes (KDIGO) CKD Work Group. KDIGO 2012 clinical practice guideline for the evaluation and management of chronic kidney disease. *Kidney Int Suppl.* (2013) 3:1–150. 10.1038/kisup.2012.73

[28] Pencina MJ D’Agostino RB Sr. D’Agostino RB Jr. Vasan RS. Evaluating the added predictive ability of a new marker: from area under the ROC curve to reclassification and beyond. *Stat Med.* (2008) 27:157–72. 10.1002/sim.2929 17569110

[29] Pencina MJ D’Agostino RB Sr. Demler OV. Novel metrics for evaluating improvement in discrimination: net reclassification and integrated discrimination improvement for normal variables and nested models. *Stat Med.* (2012) 31:101–13. 10.1002/sim.4348 22147389 PMC3341978

[30] Schurgers LJ Barreto DV Barreto FC Liabeuf S Renard C Magdeleyns EJ The circulating inactive form of matrix gla protein is a surrogate marker for vascular calcification in chronic kidney disease: a preliminary report. *Clin J Am Soc Nephrol.* (2010) 5:568–75. 10.2215/CJN.07081009 20133489 PMC2849687

[31] Keyzer CA Vermeer C Joosten MM Knapen MH Drummen NE Navis G Vitamin K status and mortality after kidney transplantation: a cohort study. *Am J Kidney Dis.* (2015) 65:474–83. 10.1053/j.ajkd.2014.09.014 25453995

[32] Riphagen IJ Keyzer CA Drummen NEA de Borst MH Beulens JWJ Gansevoort RT Prevalence and effects of functional vitamin K insufficiency: the prevend study. *Nutrients.* (2017) 9:1334. 10.3390/nu9121334 29292751 PMC5748784

[33] Jespersen T Mollehave LT Thuesen BH Skaaby T Rossing P Toft U Uncarboxylated Matrix Gla-protein: a biomarker of vitamin K status and cardiovascular risk. *Clin Biochem.* (2020) 83:49–56. 10.1016/j.clinbiochem.2020.05.005 32422228

[34] Schlieper G Westenfeld R Kruger T Cranenburg EC Magdeleyns EJ Brandenburg VM Circulating nonphosphorylated carboxylated matrix Gla protein predicts survival in ESRD. *J Am Soc Nephrol.* (2011) 22:387–95. 10.1681/ASN.2010040339 21289218 PMC3029911

[35] Luo G Ducy P McKee MD Pinero GJ Loyer E Behringer RR Spontaneous calcification of arteries and cartilage in mice lacking matrix gla protein. *Nature.* (1997) 386:78–81. 10.1038/386078a0 9052783

[36] Herrmann SM Whatling C Brand E Nicaud V Gariepy J Simon A Polymorphisms of the human matrix gla protein (MGP) gene, vascular calcification, and myocardial infarction. *Arterioscler Thromb Vasc Biol.* (2000) 20:2386–93. 10.1161/01.atv.20.11.2386 11073842

[37] Shea MK Booth SL. Concepts and controversies in evaluating vitamin K status in population-based studies. *Nutrients.* (2016) 8:8. 10.3390/nu8010008 26729160 PMC4728622

[38] Han KH O’Neill WC. Increased peripheral arterial calcification in patients receiving warfarin. *J Am Heart Assoc.* (2016) 5:e002665. 10.1161/JAHA.115.002665 26811161 PMC4859382

[39] Roumeliotis S Dounousi E Eleftheriadis T Liakopoulos V. Association of the inactive circulating matrix Gla protein with vitamin K intake, calcification, mortality, and cardiovascular disease: a review. *Int J Mol Sci.* (2019) 20:628. 10.3390/ijms20030628 30717170 PMC6387246

[40] Rennenberg RJ Schurgers LJ Vermeer C Scholte JB Houben AJ de Leeuw PW Renal handling of matrix Gla-protein in humans with moderate to severe hypertension. *Hypertens Res.* (2008) 31:1745–51. 10.1291/hypres.31.1745 18971553

[41] Wei FF Drummen NE Schutte AE Thijs L Jacobs L Petit T Vitamin K dependent protection of renal function in multi-ethnic population studies. *EBioMedicine.* (2016) 4:162–9. 10.1016/j.ebiom.2016.01.011 26981580 PMC4776057

[42] Wei FF Trenson S Thijs L Huang QF Zhang ZY Yang WY Desphospho-uncarboxylated matrix Gla protein is a novel circulating biomarker predicting deterioration of renal function in the general population. *Nephrol Dial Transplant.* (2018) 33:1122–8. 10.1093/ndt/gfx258 28992263 PMC6030862

[43] Roumeliotis S Roumeliotis A Stamou A Leivaditis K Kantartzi K Panagoutsos S The Association of dp-ucMGP with cardiovascular morbidity and decreased renal function in diabetic chronic kidney disease. *Int J Mol Sci.* (2020) 21:6035. 10.3390/ijms21176035 32839405 PMC7504709

[44] Puzantian H Akers SR Oldland G Javaid K Miller R Ge Y Circulating dephospho-uncarboxylated matrix Gla-protein is associated with kidney dysfunction and arterial stiffness. *Am J Hypertens.* (2018) 31:988–94. 10.1093/ajh/hpy079 29788226 PMC6077812

[45] Groothof D Post A Sotomayor CG Keyzer CA Flores-Guerero JL Hak E Functional vitamin K status and risk of incident chronic kidney disease and microalbuminuria: a prospective general population-based cohort study. *Nephrol Dial Transplant.* (2020) 36:2290–9. 10.1093/ndt/gfaa304 33313895 PMC8643608

[46] Fusaro M D’Alessandro C Noale M Tripepi G Plebani M Veronese N Low vitamin K1 intake in haemodialysis patients. *Clin Nutr.* (2017) 36:601–7. 10.1016/j.clnu.2016.04.024 27234935

