# Supplementary material for: Urinary matrix Gla protein is associated with mortality risk in Flemish population: A prospective study
Source: Front Cardiovasc Med. 2022 Jul 22;9:894447. doi: 10.3389/fcvm.2022.894447 (PMC9353515; doi:10.3389/fcvm.2022.894447)
Supplement: Supplementary file 1 [file Data_Sheet_1.pdf]

Supplementary Material

1 Supplementary Method

1.1 Urinary Proteomics

For proteomic analysis, a 0.7 mL aliquot of stored urine was thawed immediately before use and diluted with 0.7 mL of 2 M urea, 10 mM NH_4OH containing 0.02% sodium dodecyl sulphate. To remove higher molecular mass proteins, such as albumin and immunoglobulins, the sample was ultra-filtered, using Centrisart ultracentrifugation filter devices (20 kDa MWCO; Sartorius, Göttingen, Germany) at 3000 relative centrifugal force units until 1.1 mL of filtrate was obtained. This filtrate was then applied onto a PD-10 desalting column (GE Healthcare, Uppsala, Sweden) equilibrated in 0.01% NH_4OH in HPLC-grade in H_2O (Carl Roth GmbH, Karlsruhe, Germany) to decrease matrix effects by removing urea, electrolytes, salts, and to enrich polypeptides. Finally, all samples were lyophilized, stored at 4°C, and suspended in HPLC-grade H_2O shortly before CE-MS analyses.

Capillary electrophoresis coupled to mass spectrometry (CE-MS) was performed, using a P/ACE MDQ capillary electrophoresis system (Beckman Coulter, Fullerton, CA) on-line coupled to a micrOTOF MS (Bruker Daltonics, Bremen, Germany).[1, 2] The electrospray ionization device (Agilent Technologies, Palo Alto, CA) was grounded, and the ion spray interface potential was set between -4 and -4.5 kV. Data acquisition and MS acquisition methods were automatically controlled by the CE via contact-close-relays. Spectra were accumulated every 3 seconds over a mass-to-charge ratio (m/z) ranging from 350 to 3000.

1.2 Quality control

Accuracy, precision, selectivity, sensitivity, reproducibility and stability of the CE-MS have been previously published.[1, 3] Quality control involves daily CE-MS analysis of a human urine standard.[3] To prevent variability due to carry-over effects from one to the next analysis, capillaries are reconditioned between runs with 1 M NaOH. The coefficient of variance estimated from over 600 human urine standard analyses for over 3 years was 5.8%.[4]

1.3 Mass spectrometric data processing

Mass spectral peaks representing identical molecules at different charge states were deconvoluted into single masses, using MosaiquesVisu software.[5] Only signals with a charge > 1 observed in a minimum of three consecutive spectra with a signal-to-noise ratio of at least 4 were considered. Reference signals of 1770 urinary polypeptides were used for CE-time calibration by locally weighted regression. For normalization of analytical and urine dilution variances, signal intensities were normalized relative to 29 ''*housekeeping*'' peptides.[6, 7] The obtained peak lists characterize each polypeptide by its molecular mass. normalized CE migration time and normalized signal intensity. All detected peptides were deposited, matched, and annotated in a Microsoft SQL database, allowing further statistical analysis.[8] For clustering, peptides in different samples were considered identical, if mass deviation was less than 50 ppm. CE migration time was controlled to be below 0.35 minutes after calibration.

1.4 Sequencing of polypeptides

CE-MS signals were in silico assigned to the previously sequenced peptides from Human Urinary Proteome Database, version 2.0.[9] Peptides from this database were sequenced, as described elsewhere.[10, 11] Briefly, urinary peptides were fragmented, using different tandem mass-spectrometric techniques with a prior separation step with CE or HPLC. Fragmentation spectra were matched to the protein sequences from up-to-date public databases (IPI, NCBI Reference Sequence Database and Uniprot), using MS/MS search engines MASCOT (Matrix Sciences Ltd., London, UK) and OMSSA (National Center for Biotechnology Information, Bethesda, MD). In matching, we accounted for urinary proteins post-translational modifications, such as hydroxylation of lysine and proline, and specific MS characteristics. Peptide sequences from LC-MS/MS analyses were verified by the comparison of experimental and theoretical CE migration time, which is dependent on the number of basic and neutral polar amino acids.

1.5 Urinary MGP peptides

In 776 participants, 3 different types of MGP peptides were detected. They shared the same amino acid residues of MGP sequence 53-64. Of them, one peptide was detectable in 776 (100.0%) participants, whereas the other two peptides were found in 631 (81.3%) and 5 (0.6%) participants, respectively. The peptide (MGP sequence 54-61) with the highest prevalence was used in the analyses since these peptides had extremely similar sequences and were highly correlated with each other (Spearman's correlation coefficient ≥0.86). The detailed information is presented in Figure S1.

2 Supplementary Figure

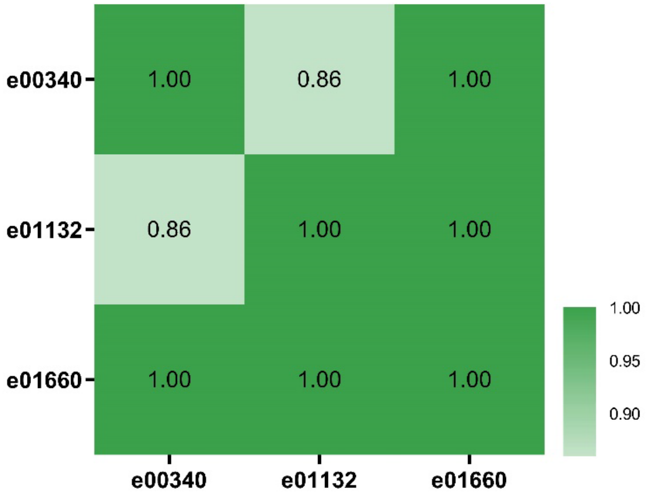

Peptide ID	Mass (Da)	CE time (min)	Sequence	Prevalence (%)
e00340	884.3	27.7	cDDYRLc	631 (81.3)
e01132	1013.4	25.1	cDDYRLcE	776 (100.0)
e01660	1084.4	29.2	AcDDYRLcE	5 (0.6)

Supplementary Figure 1. Urinary MGP peptides and their correlations. In 776 participants, 3 types of urinary peptides of matrix Gla protein (MGP) were detected. The peptides of MGP had high correlations with each other (Spearman's rank correlation coefficients ≥0.86) by using their signal intensity as a semi-quantified measurement.

3 Supplementary Table

3.1 Table S1. Participant Characteristics Across the Thirds of Plasma dp-ucMGP

Characteristics	Low (n = 258)	Medium (n = 259)	High (n = 259)	*P* for trend
Plasma dp-ucMGP (Log_2), nmol/L	<0.323	0.323–0.524	>0.524	
Number with characteristic (%)				
Female	109 (50.7)	107 (49.5)	110 (51.2)	0.004
Current Smoking	56 (26.1)	41 (19.0)	23 (10.7)	0.043
Current alcohol intake	151 (70.2)	147 (68.1)	133 (61.9)	0.049
Diabetes mellitus	3 (1.4)	10 (4.6)	16 (7.4)	0.82
History of CVD	4 (1.9)	17 (7.9)	33 (15.4)	<0.0001
Hypertension	63 (29.3)	88 (40.7)	139 (64.7)	<0.0001
Treatment of hypertension	34 (15.8)	57 (26.4)	91 (42.3)	0.0001
Statins	22 (10.2)	26 (12.0)	40 (18.6)	0.20
Warfarin	0 (0.0)	0 (0.0)	6 (2.8)	0.001
Mean (±SD) or median (IQR)				
Age, years	44.6 ± 14.4	49.9 ± 15.4	59.8 ± 13.9	<0.0001
Body mass index, kg/m^2	24.93 ± 3.90	26.1 ± 3.9	28.5 ± 4.8	<0.0001
Waist-to-hip ratio	0.8 ± 0.1	0.9 ± 0.1	0.9 ± 0.1	<0.0001
Systolic blood pressure, mmHg	124.3 ± 14.4	129.5 ± 17.6	137.3 ± 19.2	<0.0001
Diastolic blood pressure, mmHg	78.1 ± 9.5	79.8 ± 8.9	82.3 ± 10.1	<0.0001
Serum total cholesterol, mmol/L	5.08 ± 0.88	5.2 ± 0.9	5.4 ± 1.0	0.0003
HDL-cholesterol, mmol/L	1.46 ± 0.37	1.4 ± 0.4	1.4 ± 0.3	0.049
LDL-cholesterol, mmol/L	3.03 ± 0.77	3.1 ± 0.8	3.4 ± 0.9	<0.0001
Blood glucose, mmol/L	4.81 ± 0.59	5.0 ± 1.0	5.1 ± 0.9	<0.0001
Serum creatinine, mg/dL	0.89 ± 0.15	0.9 ± 0.2	1.0 ± 0.2	<0.0001
eGFR, ml/min/1.73m^2	91.9 ± 17.0	86.6 ± 17.1	76.6 ± 16.6	<0.0001
Urine albumin, mg/L	5.30 (3.90-7.10)	5.50 (3.90-7.45)	6.00 (4.30-9.30)	<0.0001
Plasma dp-ucMGP (Log_2), unit	10.21 (9.45-11.02)	10.68 (9.91-11.41)	11.31 (10.59-12.13)	0.003

Current smoking refers to inhaling tobacco daily; Diabetes mellitus was use of antidiabetic drugs, fasting blood glucose of ≥126 mg/dL; Hypertension was an office blood pressure of ≥140 mmHg systolic or ≥90 mmHg diastolic, or use of antihypertensive drugs; Body mass index was calculated by weight in kilograms divided by height in meters squared; Glomerular filtration rate was estimated using the chronic kidney disease epidemiology collaboration creatinine equation. Abbreviation: CVD, cardiovascular diseases; eGFR, estimated glomerular filtration rate; HDL, high-density lipoprotein; IQR, interquartile range; LDL, high–density lipoprotein; MGP, matrix Gla protein; SD, standard deviation.
